# Synthesis, Characterization and High *In Vitro* Antitumour Activity of Novel Triphenyltin Carboxylates

**DOI:** 10.1155/MBD.1994.305

**Published:** 1994

**Authors:** Marcel Gielen, Abdelaziz El Khloufi, Monique Biesemans, Abdeslam Bouhdid, Dick de Vos, Bernard Mahieu, Rudolph Willem

**Affiliations:** 1 Free University of Brussels (V.U.B.) Pleinlaan 2 Faculty of Applied Sciences Laboratory for General and Organic Chemistry (AOSC), Room 8G512 Brussels B-1050 Belgium; 2 Free University of Brussels (V.U.B.) Pleinlaan 2 High Resolution NMR Center Brussels B-1050 Belgium; 3 Free University of Brussels (V.U.B.) Pleinlaan 2 Faculty of Sciences Brussels B-1050 Belgium; 4 Medical Department Pharmachemie BV RN Haarlem NL-2003 The Netherlands; 5 Catholic University of Louvain INAN Louvain-la-Neuve B-1348 Belgium

## Abstract

The synthesis and spectral characterization of six novel triphenyltin compounds are described. The *in vitro* antitumour activity of three of these compounds against two human tumour cell lines, MCF-7, a mammary tumour, and WiDr, a colon carcinoma, was determined. All three compounds are more active than cis-platin, etoposide and doxorubicin against both tumour cell lines. They are as active as mitomycin C against WiDr, but less active against MCF-7.


					SYNTHESIS, CHARACTERIZATION AND
HIGH IN VITRO ANTITUMOUR ACTIVITY OF
NOVEL TRIPHENYLTIN CARBOXYLATES
Marcel Gielena, Abdelaziz El Khloufic, Monique Biesemansla, lb,
Abdeslam Bouhdidc, Dick de Vos2, Bernard Mahieu3 and Rudolph Willema, b
Free University of Brussels (V.U.B.), Pleinlaan 2, B-1050 Brussels, Belgium
a Faculty of Applied Sciences, Laboratory for General and Organic Chemistry (AOSC), Room 8G512
b High Resolution NMR Center c Faculty of Sciences
2 Medical Department, Pharmachemie BV, NL-2003 RN Haarlem, The Netherlands
3 Catholic University of Louvain, INAN, B-1348 Louvain-la-Neuve, Belgium
Abstract
The synthesis and spectral characterization of six novel triphenyltin compounds are described. The
in vitro antitumour activity of three of these compounds against two human tumour cell lines, MCF-7,
a mammary tumour, and WiDr, a colon carcinoma, was determined. All three compounds are more
active than cis-platin, etoposide and doxorubicin against both tumour cell lines. They are as active
as mitomycin C against WiDr, but less active against MCF-7.
Introduction
The exceptionally high in vitro antitumour activity of a series of substituted triphenyltin benzoates(I)
against MCF-7, a mammary tumour (6 18 ng/ml) and WiDr, a colon carcinoma (15 18 ng/ml)
prompted us to prepare additional triphenyltin compounds in order to examine whether the nature
and substitution pattern of the aromatic ring of the carboxylate moiety influences specifically their
activity.
Results and discussion
Synthesis
The substituted triphenyltin benzoates were synthesized by a 1:1 condensation of triphenyltin
hydroxide with the appropriate substituted benzoic acids(1).
XYZC6H2CO2H + Ph3SnOH XYZC6H2CO2SnPh3 + H20
1: 3-FC6H4CO2SnPh3
2: 2,3-F2C6H3CO2SnPh3
3: 2,3,4-(CH30)3C6H2CO2SnPh3
4: 2,4,5-(CH30)3C6H2CO2SnPh3
5: 3,4,5-(CH30)3C6H2CO2SnPh3
305
VoL 1. No. 4. 1994 Synthesis, Characterization andHtgh in vitroAntitumourActivity
ofNovel Triphenyltin Carboxylates
Ph
O------Sn
COOH Ph3SnOH
O
$-CH3 S.CH3
Ph
O m
Spectroscopic characterization
The spectral data characterizing compounds 1 to 6 are given in the experimental part.
The isomer shift (IS, in the range 1.12 1.28 mm/s) and quadrupole splitting (QS, in the range 2.25
2.46 mm/s) of the triphenyltin benzoates are in agreement with tetrahedral geometries(2) at the tin
atom (see figure 1).
Ph
O
ph,,,,,,,t.:oSn
Ph
Figure 1: Tetrahedral geometry proposed for the substituted
triphenyltin benzoates, XYZC6H2CO2SnPh3
The nj(13C, 19F) and nj(13C, 119/117Sn) couplings enabled an easy assignment of the carbon
atoms of the benzoate respectively phenyl groups. Where necessary, in the absence of fluorine,
13C DEPT spectra were used, together with aromatic 13C chemical shift increments.
The values of the 1j(13C, 119/117Sn coupling constants around 650/620 Hz and of the 119Sn NMR
chemical shifts in the range -100 to -120 ppm suggest the triphenyltin carboxylates to exhibit a
tetracoordinated geometry(3-5) in solution like in the solid state.
In vitro antitumour tests
The results of the in vitro activity tests against two human tumour cell lines, MCF-7, a mammary
tumour, and WiDr, a colon carcinoma, performed on compounds 2, 3 and 5, are presented in table
1.
They are of comparable activity as the previously described triphenyltin carboxylates(1), i.e. more
active than cis-platin, etoposide and doxorubicin against both tumour cell lines. They are as active
as mitomycin C against WiDr but less active against MCF-7.
306
M. Giln, .ElKhloufl, M. Btsmans, A. Bouhdid, D. da Voa, B. Mahtau andR. Willam MetalBasedDrugs
Compound
MCF-7
Cell lines
WiDr
:2, 2,3-F2C6H3CO2SnPh3
:3, 2,4,5-(CH30)3C6H2CO2SnPh3
5, 2-SCH3-3-C5NH3CO2SnPh3
17
16
16
24
15
18
Cis-platin(6) 850 624
Etoposde(6) 187 62
Doxorubicin(6) 63 31
Mitomycin C(6) 3 1
Table 1: Inhibition doses ID50 in ng/mL against two human tumour cell lines, MCF-7 and WiDr,
obtained for compounds 2, 3 and 5, and for four clinically used antitumour drugs(6)
Experimental part
Syntheses and spectral characterization
Equimolar amounts of triphenyltin hydroxide and substituted benzoic acid are poured into a three-
necked 250 mL flask equipped with a Dean-Stark funnel containing 200 mL of a toluene/ethanol
3/1 mixture. The mixture is refluxed for 4 h. The ternary azeotrope water/ethanol/toluene followed
by the binary azeotrope toluene/ethanol are distilled off to 50% of the initial volume. The remaining
solution is evaporated under reduced pressure. The solid obtained is recrystallised from the
appropriate solvent.
3
Ph
Compound 1, triphenyltin 3-fluorobenzoate, mp (recryst. from n-hexane): 89-90 C, yield: 46%,
Mssbauer parameters: quadrupole splitting QS: 2.44, isomer shift IS: 1.25, band widths: 0.91 and
0.93 mm/s; 1H NMR (chemical shifts in ppm with respect to TMS; nj(1H, 1H) coupling constants in
Hz between parentheses; nj(1H, 19F) coupling constants in bold)" 7.91 ppm, dd (8,1)" H(2); 7.20,
dddd (8,8,3,1): H(4); 7.36, ddd (8,8,6): H(5); 7.76-7.82, m: H(6) and H(o); 7.41-7.51, m: H(m) +
H(p); 19F NMR: -113.8, ddd" (9,9,6); 13C NMR (nj(13C,19F) coupling constants in Hz between
parentheses; nj(13C,119/117Sn) coupling constants in Hz in bold between parentheses;
calculated chemical shifts(7)(8) between brackets)" 133.1 [132.0], d (3j 7): C(1); 117.3 [117.1], d
(2j 23)" C(2); 162.4 [163.3], d (1j 247)" C(3); 119.5 [120.7], d (2j 21): C(4); 129.6 [129.9], d
307
Vol. 1, No. 4, 1994 Synthesis, Characterization andHigh in vitroAntitumourActivity
ofNovel Triphenyltin Carboxylates
(3j 8)" C(5); 126.2 [125.5], d (4j 2): C(6); 171.3, s: C(7); 138.1, s (1j 648/620)" C(i); 136.8, s
(unresolved 2j 48): C(o); 128.9, s (unresolved 3j 64): C(m); 132.2, s: C(p); 19Sn NMR:
-106.3.
Compound 2, triphenyltin 2,3-difluorobenzoate, mp (recryst. from ethanol): 87-89 C, yield: 81%,
M6ssbauer parameters: quadrupole splitting QS: 2.48, isomer shift IS: 1.25, band widths: 0.91 and
0.91 mm/s; 1H NMR, chemical shifts in ppm with respect to TMS, nj(1H, 1H) coupling constants in
Hz between parentheses (nj(1H, 19F) coupling constants in bold): 6.58, dddd (11,10,7,2):
H(4);6.33, dddd (10,10,5,2): H(5); 7.22, dddd (10,6,3,2): H(6); 7.83-7.86, m: H(o); 7.09-7.23, m:
H(m) + H(p); 19F NMR, chemical shifts with respect to CFCI3: -134.5, (o-F); -137.8, (m-F), broad
singlets; 13C NMR, chemical shift in ppm with respect to TMS, (nj(13C,1"9F) coupling constants in Hz
between parentheses; nj(13C,119/117Sn coupling constants in Hz in bold between parentheses;
calculated chemical shifts(7)(8) between brackets)" 121.4 [119.1], b: C(1); 151.2 [151.9], dd (1j
247, 2j 13): C(2); 151.0 [150.4], dd (1j 260, 2j 137)" C(3); 120.8 [122.1], d (2j 18): C(4);
123.2 [125.4], dd (3j & 4j 6 & 5)" C(5); 126.9 [126.95], b: C(6); 169.3, s: C(7); 138.2, s (1j
647/617)" C(i); 137.1, s (unresolved 2j 49): C(o); 129.3, s (3j 65/62)" C(m); 130.3, s: C(p);
119Sn NMR: -102.1 (1j(119Sn,13C) 647).
Compound 3, triphenyltin 2,3,4-trimethoxybenzoate, mp (recryst. from chloroform/n-hexane): 82-
83 C, yield: 84%; M6ssbauer parameters: QS: 2.25, IS: 1.12, band widths: 1.32 and 1.35; 1H NMR
(estimated 3j(1H,117/119Sn coupling constants from non resolved satellites between brackets in
bold): 6.66, d (9): H(5); 7.78, d (9): H(6); 7.79-7.83, rn [=60]: H(o); 7.38-7.47, m: H(m) + H(p); 3.85,
3.86 and 3.88: CH30; 13C NMR: 117.7 [109.1]: C(1); 155.2 and 157.2 [148.4 and 151.0]: C(2) and
C(4); 142.9 [130.8]" C(3); 106.8 [107.1]" C(5); 128.3 [124.7]: C(6); 172.0: C(7); 138.8 (1j
649/620): C(i); 136.9 (unresolved 2j 48)" C(o); 128.8 (unresolved 3j 63): C(m); 130.0: C(p);
56.0, 61.0 and 61.8: CH30; 119Sn NMR:-118.0.
Compound 4, triphenyltin 2,4,5-trimethoxybenzoate, mp (recryst. from chloroform/n-hexane): 135-
136 C, yield" 94%; M6ssbauer parameters: QS: 2.29, IS: 1.24, band widths: 0.81 and 0.86; 1H
NMR: 6.51, s: H(3); 7.56, s: H(6); 7.77-7.82, m [=66]: H(o); 7.39-7.4.6, m: H(m) + H(p); 3.86, 3.88
and 3.91" CH30; 13C NMR: 111.0 [109.1]" C(1); 156.1 and 153.6 [155.1 and 151.0]" C(2) and C(4);
98.3 [100.4]" C(3); 142.6 [137.5]" C(5); 115.9 [118.0]" C(6); 171.9" C(7); 138.9 (1j 649/620)"
C(i); 136.9 (2j 48): C(o); 128.7 (3j 63): C(m); 129.9: C(p); 55.9, 56.4 and 57.4: CH30; 119Sn
NMR: -120.4.
Compound 5, triphenyltin 3,4,5-trimethoxybenzoate, mp (recryst. from ethanol): 114-115 C, yield:
91%; M6ssbauer parameters" QS" 2.35, IS: 1.22, band widths: 0.88 and 0.95; 1H NMR: 7.32, s:
H(2) H(6); 7.78-7.81, m [--62]: H(o); 7.42-7.49, m: H(m) + H(p); 3.87:3-CH30 5-CH30; 3.88: 4-
CH30; 13C NMR: 125.5 [124.5]: C(1); 108.0 [109.3]" C(2) C(6); 152.7 [146.2]: C(3) C(5); 128.2
[135.6]" C(4); 172.4:.C(7); 138.4 (1j 649/620). C(i); 136.8 (2j 48): C(o); 128.9 (3j 63)" C(m);
130.1" C(p); 60.8" 3-CH30 5-CH30; 56.2: 4-CH30; 119Sn NMR:-112.4.
Compound 6, triphenyltin 2-methylthio-3-pyridinecarboxylate, mp (recryst. from ethanol): 130-
133 C, yield" 71%; M6ssbauer parameters: QS: 2.46, IS: 1.28, band widths: 0.86 and 0.90; 1H
NMR" 8.32, dd (8,2): H(4); 7.01, dd (8,5): H(5); 8.53, dd (5,2): H(6); 7.80- 7.83, m [3j 66]" H(o);
308
M. Gtelen, A. ElKhloufl, M. Btescmans, A. Bouhdid, D. de Vos, B. Mahteu andR. Willem MetalBasedDrugs
7.45 7.50, m: H(m + p); 2.52, s: SCH3; 13C NMR: 162.8 [chemical shift calculated with the chemical
shift increments characteristic for pyridine systems(7)(8): 161.4]: C(2); 124.0 [124.5]: C(3); 139.9
[138.1]" C(4); 117.7 [120.6]: C(5); 151.5 [155.2]" C(6); 171.3: C(7); 138.1 (1j 647/618): C(i);
136.9 (2j 49): C(o); 128.9 (3j = 64)" C(m); 130.1" C(p); 13.9: SCH3; 119Sn NMR:-102.4.
References
(1) Gielen M, Willem R, Biesemans M, Bou&lam M, El Khloufi A, and de Vos D, AppL
Organomet. Chem., 6 (1992), 287
(2) Zuckerman JJ, in "Chemical MSssbauer Spectroscopy", Ed.: Herber RH, Plenum Press,
New York (1984), p. 167
(3)
(4)
(5)
(6)
(7)
(8)
Holecek J, Nadvornik M, Handlir K, and Lycka A, J. Organomet. Chem., 241 (1983) 177
Nadvornik M, Holecek J, Handlir K and Lycka A, J. Organomet. Chem., 275 (1984) 43
Sandhu GK, Kaur G, Holecek J, and Lycka A, J. Organomet. Chem., 365 (1989) 215
van Lambalgen R and Lelieveld P, Invest. New Drugs, 5 (1987), 161
Kalinowski HO, Berger S, and Braun S, "Carbon NMR Spectroscopy", J. Wiley, Chichester,
(1988), p. 313
Bou&lam M, Willem R, Biesemans M, and Gielen M, AppL Organomet. Chem., 5 (1991) 497
Received" January 17, 1994 Accepted: February 14, 1994 Received in
revised camera-ready format: February 18, 1994
309

				

